# An ultra-late maxillofacial cavity following multimodal treatment for adult head and neck rhabdomyosarcoma: a case report with a 16-year follow-up

**DOI:** 10.3389/fonc.2026.1765985

**Published:** 2026-06-29

**Authors:** Wanhui Dong, Sheng Xu, Li Cheng, Jing Xu, Bin Xu, Zhaidong Liu

**Affiliations:** 1 Shandong University of Traditional Chinese Medicine, Jinan, Shandong, China; 2 Department of Oncology, Anhui University of Traditional Chinese Medicine Affiliated Lu'an Traditional Chinese Medicine Hospital, Lu’an, China; 3 Department of Oncology, Shandong University of Traditional Chinese Medicine Affiliated Hospital, Jinan, Shandong, China

**Keywords:** head and neck neoplasms, maxillofacial cavity, osteonecrosis, radiation injuries, rhabdomyosarcoma

## Abstract

**Objective:**

To report a rare case of a patient with adult rhabdomyosarcoma (RMS) in the craniofacial region who developed a very late-stage left maxillofacial cavity after 16 years of multimodal treatment, and to systematically investigate the potential causal relationship between this complication and previous radiotherapy.

**Methods:**

A detailed report is presented on the diagnosis and treatment process of a 46-year-old female patient. A comprehensive evaluation framework was employed to analyze causality, encompassing temporal correlation, spatial alignment with the original radiation field, exclusion of alternative etiologies, and biological plausibility.

**Results:**

Approximately 16 years after the initial radiotherapy, the patient developed soft tissue defects and cavity formation in the core area of the original high-dose irradiation field. Although tissue biopsy was not performed due to post-radiotherapy site bleeding and patient constitution risks, imaging studies showed a high degree of consistency between the lesion and the previous radiotherapy area, with no evident evidence of tumor recurrence or primary infection, consistent with the pathophysiological process of radiation-induced tissue necrosis.

**Conclusion:**

Despite the exceedingly long latency period, based on the conclusive spatial correlation, reasonable exclusion of other etiologies, and well-established radiobiological mechanisms, there is a “highly probable” causal relationship between the maxillofacial cavity and previous radiotherapy. This case serves as a warning that for long-term surviving RMS patients, extremely late radiation injury represents a serious complication risk that cannot be ignored.

## 1 Introduction

Rhabdomyosarcoma (RMS) is the most common soft tissue sarcoma in children and adolescents (1), accounting for approximately 7% of all malignant tumors and 50% of soft tissue sarcomas in this population (2). However, its incidence in adults is extremely low, representing for less than 1% of all adult malignancies (3). Although the head and face region is one of the most common sites for RMS in children, its occurrence in adults is particularly rare, resulting limited research focused on this specific population (4). Most diagnostic and treatment protocols are based on pediatric data, with management relying on multimodal strategies, including surgery, radiotherapy, and chemotherapy. While radiotherapy is essential for local tumor control, its long-term toxicities-such as radiation-induced osteonecrosis and tissue fibrosis—pose significant challenges to the quality of life of long-term survivors (5, 6). Typically, radiotherapy-related injuries manifest within months to several years after treatment. However, the etiology of severe tissue defects arising more than ten years post-radiotherapy is highly complex, presenting substantial clinical challenges in diagnosis and attribution.

This case involves an adult with head and face RMS spanning 16 years, which eventually developed into left maxillofacial cavity. The primary objective of this report is not to assert a single cause but to rigorously explore the causal association between this very late complication and the likelihood of previous radiotherapy by systematically collecting and presenting evidence. We will follow a structured analytical framework, examining four dimensions: temporal dynamics, spatial consistency, exclusion of alternative diagnoses, and biological plausibility, to construct the chain of reasoning step by step. This case highlights the critical importance of understanding and managing very late therapeutic toxicities in the pursuit of long-term tumor control and provides a valuable analytical paradigm and decision-making reference for clinicians evaluating similarly complex situations.

## 2 Case presentation

The patient is a 46-year-old Han Chinese female (height 154 cm, weight 43 kg, BMI 18.1 kg/m², KPS score 50) with no history of smoking, alcohol consumption, malignant tumors, or familial hereditary tumor syndromes, and no occupational radiation exposure. Due to limited conditions at the initial diagnosis, PAX3/7-FOXO1 fusion or MYOD1 mutation gene testing was not performed. In September 2009, the patient underwent ‘ left maxillary tumor resection + left maxillary subtotal resection + external carotid artery ligation ‘ because of the left maxillary tumor (**Figure 1**). Postoperative pathological and immunohistochemical results showed that Desmin was positive and Myogenic Differentiation 1(MyoD1) was also positive. Steroidogenic Factor 1(SF-1) +, Ki-67 +, diagnosed as RMS (**Figure 2**). The patient had no history of diabetes and hypertension. There is no history of malignant tumors (including soft tissue sarcomas) in first-degree relatives (parents, siblings, children), nor is there a family history of hereditary tumor syndromes (such as Li-Fraumeni syndrome, neurofibromatosis).

**Figure 1:**
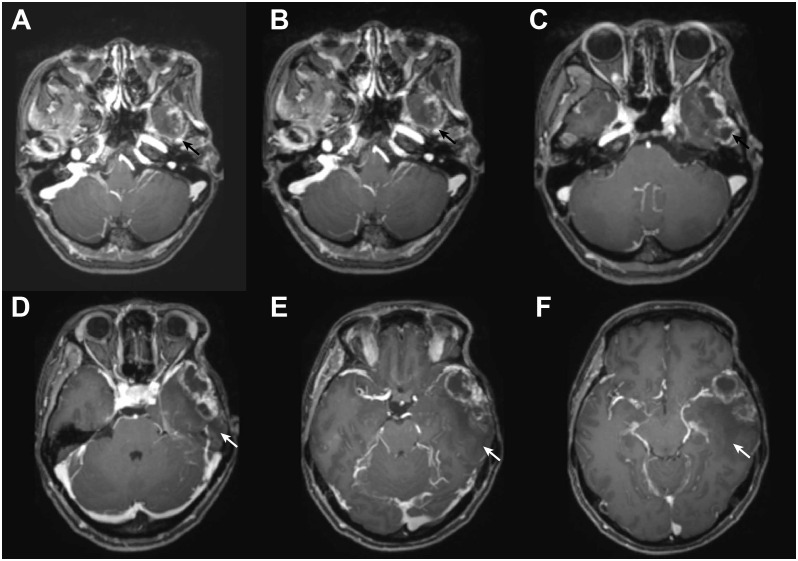
Enhanced MRI images of patients at the first diagnosis and treatment. **(A–C)** Axial images show multiple irregular, rosette-like enhancing lesions (black arrows) in the left temporal lobe, with central non-enhancing areas. **(D–F)** Surrounding brain parenchyma demonstrates extensive patchy edema (white arrows). The interface with adjacent meninges is ill-defined, and focal bone destruction of the skull base is noted. No additional parenchymal enhancement is observed. Midline structures are preserved, and no ventricular dilatation is present.

**Figure 2:**
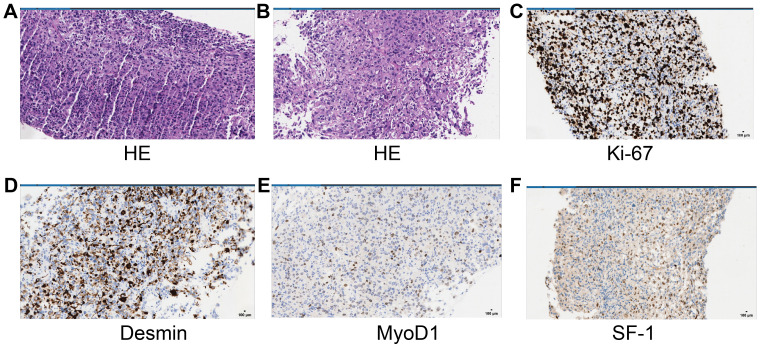
Immunohistochemical images of patients after the first operation. **(A)** HE staining showed that the tumor cells were significantly atypia, morphologically diverse, with different nuclear sizes, darkly stained chromatin, and diffuse disordered arrangement. **(B)** The tumor cells exhibit both diffuse and focal distribution, with brown granular positive staining visible within the cytoplasm (black arrow). **(C–F)** Ki-67, Desmin, MyoD1 and SF-1 were all positive (black arrows). (The magnification is 10×20, and the scale is 100μm).

Due to disease recurrence, the patient underwent a total of six surgeries, multiple courses of chemotherapy and two rounds of radiotherapy (**Table 1**).

**Table 1:** Patient treatment timeline and clinical course.

Date	Intervention type	Details	Notes/reason for adjustment
September 2009	Surgery	Left maxillary tumor resection + left maxillary subtotal resection + external carotid artery ligation	Initial surgery; pathological diagnosis of rhabdomyosarcoma
2009–2013	Radiotherapy	**First course**: 50 Gy/25 fractions **Second course**: 34 Gy/15 fractions	Two courses delivered due to disease recurrence; cumulative dose approximately 84Gy
2009–2013	Surgery	Six surgeries in total due to recurrent disease	Last surgery: October 29, 2013
February 2024	MDT Decision	Multidisciplinary team discussion concluded no surgical indication	Transition to medical oncology management
May 3–24, 2024	Chemotherapy	IE regimen: IFO 2.7g d1-3 + VP-16 150mg d1	2 cycles
June 16, 2024	Chemotherapy	VAC regimen: VCR 2mg d1 + ADM 75mg d1 + CTX 1.1g d1	Switched due to grade III bone marrow suppression
July 12, 2024	Chemotherapy	IE regimen (same dosage)	1 cycle
August 2, 2024	Chemotherapy + Targeted	VAC regimen + anlotinib	Combined targeted therapy
August 23, 2024	Chemotherapy + Targeted	IE regimen + anlotinib	Continued combined therapy
October 10, 2024	MDT Decision	Second MDT discussion, no surgical indication	Continue medical management
October 19 – November 18, 2024	Chemotherapy + Targeted	VCR 2mg d1 + epirubicin 50mg d1 + anlotinib 10mg d3-16	2 cycles
December 31, 2024	Chemotherapy + Targeted	VCR 2mg d1 + liposomal irinotecan 37.66mg d1 + anlotinib 10mg d3-16	Adjusted due to bone marrow suppression
January 21, 2025	Targeted Maintenance	Chemotherapy suspended; continued oral anlotinib	Due to bone marrow suppression
February 24, 2025	Chemotherapy + Targeted	VCR + liposomal irinotecan + anlotinib	Treatment resumed
March 17, 2025	Chemotherapy + Targeted	VCR 2mg d1 + epirubicin 50mg d1 + anlotinib 10mg d2-15	Adjusted due to severe systemic adverse reactions
April 15, 2025	Chemotherapy + Targeted	VCR 2mg d1 + liposomal irinotecan 37.66mg d1 + bevacizumab 700mg	Targeted agent switched due to severe oral mucositis
May 16 – June 9, 2025	Chemotherapy + Targeted	VAC regimen + anlotinib	2 cycles
July 8, 2025	Chemotherapy + Targeted	VCR + liposomal irinotecan + anlotinib	1 cycle
Late July 2025	Complication	Ulceration in left temporal and maxillofacial region, progressive cavity formation	Manifestation of ultra-late radiation injury
October 16, 2025	Nutritional Assessment	Gastrostomy recommended by external hospital	Patient refused

(First course: 1st radiation treatment plan; Second course: 2st radiation treatment plan).

In late July 2025, the patient developed ulceration in the left temporal and maxillofacial regions, with the ulcer area gradually increasing. On October 16, 2025, due to poor nutritional status, an external hospital noted severe bone destruction in the maxillofacial region and recommended gastrostomy to prevent infection exacerbation during eating, but the patient declined. Upon admission, a cavity approximately 20mm × 18mm in size was observed in the left temporal region, exposing the base of the skull and maxilla deep within the cavity (**Figure 3**). Magnetic resonance imaging (MRI) revealed a local skin and soft tissue defect in the left maxillofacial region with cavity formation, disorganized structure in the surgical area, and extensive edema-like changes in the surrounding soft tissue, without clear evidence of tumor-like changes (**Figure 4**). Considering that performing invasive biopsy within a radiation field that already exhibits severe microvascular damage and ischemia is highly susceptible to induce or exacerbate radiation osteonecrosis, leading to non-healing wounds, massive hemorrhage, or secondary severe infections (7), and given the patient’s poor nutritional status and quality of life at the time, even if the pathological diagnosis clearly indicates tumor recurrence, it is impossible to implement any anti-tumor treatment interventions. Therefore, based on the multidisciplinary consultation, a comprehensive clinical-radiological diagnosis was adopted. Following multidisciplinary consultation, the patient was transitioned to best supportive care, focusing on wound debridement, local antiseptic irrigation, nutritional rehabilitation, and analgesia. Despite conservative measures, the maxillofacial cavity persisted and progressively enlarged, with ongoing exposure of the skull base and maxillary bone. The patient subsequently received intermittent intravenous infusions in the community, with their children performing daily local rinsing with saline solution and covering the surface with sterile gauze at the maxillofacial cavities, remaining in a state of chronic malnutrition. The last telephone follow-up was conducted in April 2026. This outcome underscores the devastating nature of ultra-late radiation injury in long-term survivors and the limited therapeutic options once extensive soft tissue and osteonecrosis have developed.

**Figure 3:**
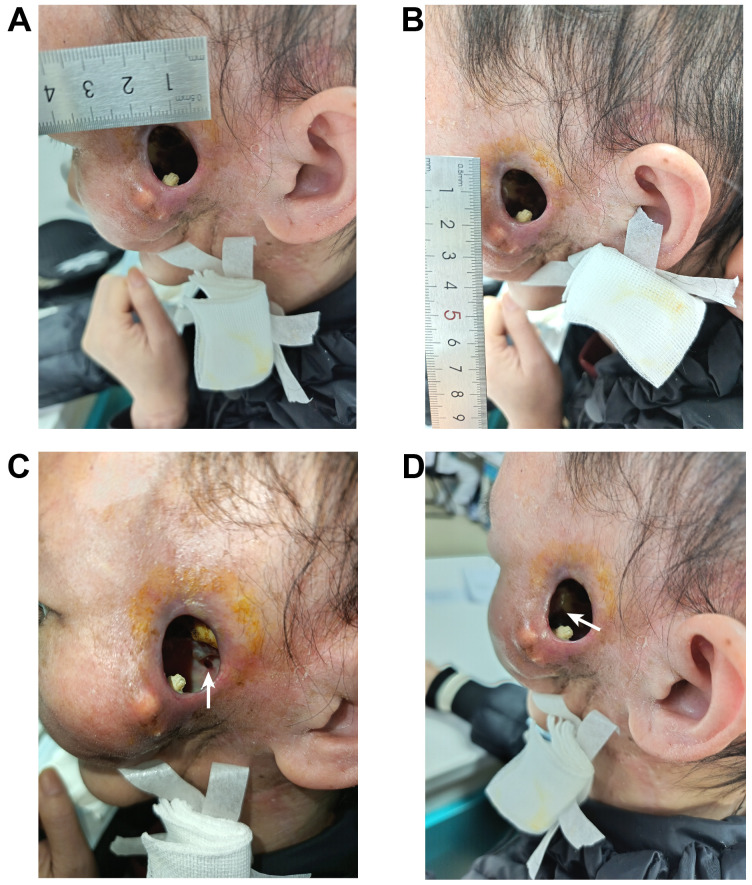
Clinical photographs demonstrating left-sided maxillofacial cavity formation. **(A, B)** Clinical photographs show a cavity measuring approximately 20 mm × 18 mm in the left temporal region. **(C, D)** Deep within the cavity, the base of the skull and maxillary bone are visible (white arrows).

**Figure 4:**
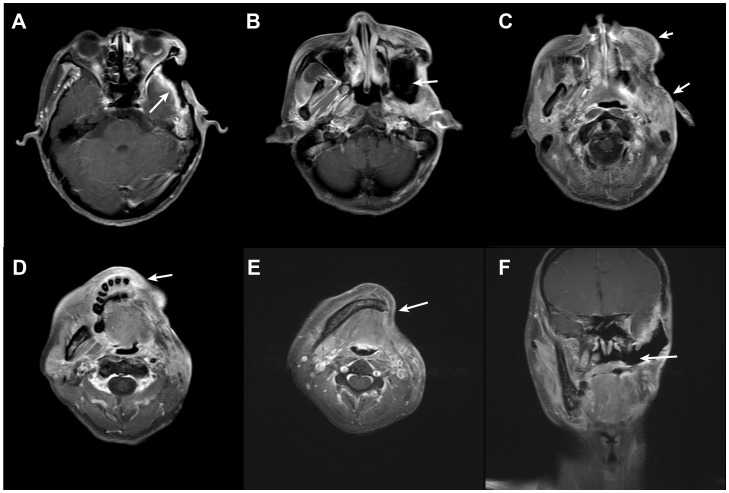
Post-contrast head MRI findings at the time of left-sided cavity presentation. **(A)** Axial image demonstrates abnormal signal in the left temporal lobe with surrounding brain parenchymal edema and adjacent meningeal thickening and enhancement (white arrow), consistent with post-treatment changes. **(B)** Coronal image reveals a localized skin and soft tissue defect with cavity formation in the left maxillofacial region (white arrow). **(C)** Structural disorganization in the operative field with extensive surrounding soft tissue edema (white arrow). **(D)** Tubular abnormal enhancement in the left upper alveolar region with associated alveolar bone defect (white arrow). **(E)** Inferior alveolar bone defect (white arrow). **(F)** left-sided maxillofacial cavity extends inward and downward to the left sided parapharyngeal space and upward to the anterior cranial fossa on left side (white arrow).

## 3 Discussion

This case report follows the CARE guidelines and describes a case of adult head and face RMS with a course of up to 16 years. After undergoing multiple surgeries, two courses of radiotherapy (with a cumulative dose of approximately 84 Gy), and multiline chemotherapy, the patient developed a huge cavity in the left maxillofacial region 16 years after completing radiotherapy. Her long-term treatment history and extremely late-stage severe complications provide valuable clinical insights into this rare disease. Based on existing literature, we closely examined the unique characteristics of this case and conducted an in-depth analysis of its clinical value and significance from multiple perspectives.

### 3.1 Diagnosis and treatment challenges of adult craniofacial RMS: typicality and specificity of this case

Adult head and face RMS is extremely rare, accounting for only about 1% of all adult soft tissue sarcomas. Compared with pediatric patients, adult RMS has unique biological characteristics (8): Pediatric patients are dominated by embryonic and alveolar rhabdomyosarcoma subtypes. In adults, the proportion of polymorphic RMS is higher, the stage at diagnosis is later, and the prognosis is significantly worse (9). The patient was a 46-year-old woman with a survival time of 16 years after diagnosis, far exceeding the average survival time of adult RMS (10, 11), which made this case a valuable example for studying the long-term disease evolution of RMS.

It is worth noting that the efficacy of adult RMS on standard chemotherapy regimens is often inferior to that of pediatric patients (12). The patient in this case received a variety of regimens such as VAC, IE, but all had to adjust the treatment due to III-degree bone marrow suppression or disease progression.

Although multimodal systemic therapy is the standard intervention strategy for adult RMS, achieving durable disease control remains extremely challenging. This clinical reality profoundly reveals the fundamental dilemma of current systemic drug therapy for adult RMS: the improvement of antitumor efficacy often comes at the significant expense of long-term quality of life. This also explains why the patient ultimately had to make a difficult trade-off between tumor control and quality of life.

### 3.2 Etiological analysis of maxillofacial cavity lesions: the rationality of radiation necrosis

In the case of a maxillofacial cavity that emerged 16 years after the completion of radiotherapy, establishing the etiology serves as the cornerstone of clinical decision-making. Although no pathological biopsy was conducted in this case, through systematic evaluation, we believe that radiation necrosis is the most probable etiology.

#### 3.2.1 Temporal correlation: extremely long latency period yet conforming to radiobiological principles

The latency period of radiation injury exhibits high individual heterogeneity. Radiation-induced osteonecrosis commonly occurs 1–5 years after treatment (13, 14), but progressive microvascular damage and fibrosis represent a chronic biological process that evolves continuously. As tissue regeneration and repair capabilities gradually decline over time, such latent injuries may manifest as clinically significant events for the first time after more than a decade. There are indeed reports in the literature of severe radiation-induced tissue damage occurring up to 8 years after radiotherapy (15), especially in patients who underwent high-dose radiotherapy and had impaired local blood supply (16). In the present case, the cumulative dose of radiotherapy administered to the patient was approximately 84 Gy, and multiple surgeries further disrupted the blood supply to the maxillofacial region. Therefore, although a latency period of 16 years is rare, it still aligns with the basic principle of “dose-time-tissue response” in radiobiology and falls within the realm of radiobiological possibility.

#### 3.2.2 Spatial correlation: conclusive evidence

This provides the strongest evidence in support of radiation etiology. The patient’s MRI imaging clearly demonstrates that the soft tissue defect cavity and surrounding changes are strictly confined to the left maxillary region, temporal region, and skull base area, which have historically received a cumulative high-dose radiation of approximately 84 Gy. The high degree of consistency in the anatomical extent of this lesion with the original radiotherapy planning area in three-dimensional space is a typical characteristic that distinguishes radiation injury from tumor recurrence or primary infection.

#### 3.2.3 Exclusion analysis: systematically eliminating competing diagnoses

Tumor recurrence: This serves as the primary distinguishing factor. However, the MRI of this case reveals that the core lesion area “shows no significant enhancement,” which is inconsistent with the typical manifestations of active, hypervascular tumors. Additionally, the patient’s overall condition prior to this event does not align with the characteristics of rapid tumor progression. Nevertheless, the absence of histopathological evidence from biopsy remains a critical limitation in this case, preventing us from completely excluding the presence of low-grade tumor activity or heterogeneous components.

Infection: There are no clinical signs of acute suppurative infection. Although MRI suggests “infectious lesions cannot be ruled out,” there is a lack of systematic evidence supporting invasive infection. Furthermore, infection is more likely to be a secondary issue that complicates on the basis of radioactive necrotic tissue, rather than being the primary cause.

#### 3.2.4 Biological rationality

This clinical presentation is fully consistent with the established pathophysiological mechanism of radiation necrosis. Radiotherapy induces chronic oxidative stress and fibrosis (17), leading to progressive vascular endothelial damage, luminal occlusion, and tissue hypoxia, ultimately triggering sterile necrosis (18).

Based on the above structured assessment, we have concluded that the maxillofacial cavity is a late-stage radiation injury (CTCAE v6.0 classification: *https://dctd.cancer.gov/research/ctep-trials/trial-development*), and there is a very likely causal relationship with the previous radiotherapy.

#### 3.2.5 Current treatment standards for adult head and neck RMS and their applicability to this case

The management of adult head and neck RMS remains challenging due to the rarity of the disease and the absence of dedicated adult-specific protocols. Currently, treatment recommendations are largely extrapolated from pediatric guidelines, with multimodal therapy comprising surgery, radiotherapy, and chemotherapy serving as the cornerstone of management. According to the FaR-RMS (Front-line and Relapsed Rhabdomyosarcoma) radiotherapy guidelines, a simultaneous integrated boost to 41.4 Gy in 23 fractions is recommended for the primary tumor, with an optional boost for residual disease (19). A recent retrospective analysis of 72 adults with head and neck RMS demonstrated that radiotherapy significantly improved overall survival (OS), progression-free survival (PFS), local recurrence-free survival (LRFS), and distant metastasis-free survival (DMFS), with optimal outcomes observed at approximately 62.5 Gy and 8 cycles of chemotherapy (20). Surgery, conversely, showed no significant survival benefit in this cohort, reflecting the anatomical constraints and aggressive biology of adult disease. In the present case, the patient received two courses of radiotherapy (50 Gy and 34 Gy) over a 4-year interval, with a cumulative dose of approximately 84 Gy—far exceeding the standard recommendations.

By contemporary standards, such a cumulative dose would be considered prohibitively high for the maxillofacial region, and modern practice would prioritize highly conformal techniques such as intensity-modulated radiotherapy (IMRT) or proton beam therapy (PBT) to maximize target coverage while sparing adjacent critical structures. PBT, in particular, has demonstrated favorable local control (84% at 5 years) and reduced late toxicity rates (26% versus 48-65% with conventional photon RT) in parameningeal RMS, primarily by eliminating the exit dose to contralateral structures and reducing integral dose to soft tissues (19, 21).

Long-term follow-up data from the Cooperative Weichteilsarkom Studiengruppe (CWS) trials demonstrate that cosmetic and functional defects are less frequently reported after proton therapy compared with conventional fractionated photon RT or hyperfractionated accelerated RT (22). For this patient, the historical use of conventional external beam radiotherapy without modern dose constraints likely resulted in a significantly higher integral dose to the maxillary bone, temporal soft tissues, and skull base, creating the biological substrate for the ultra-late necrosis observed.

Regarding systemic therapy, the patient sequentially received VAC, IE, and multiple anthracycline- or irinotecan-based regimens combined with anlotinib. While these regimens reflect the current standard of care for adult RMS, the response was transient, with repeated episodes of grade III bone marrow suppression necessitating dose reductions and treatment switches. This pattern aligns with the well-documented chemoresistance of adult RMS. In the modern era, patients with relapsed or refractory RMS are increasingly being considered for clinical trials investigating CAR-T cell therapy targeting FGFR4 or EPHB4, immune checkpoint inhibitors, and molecularly matched targeted agents. However, for this patient, whose disease course was dominated by cumulative local toxicity rather than active metastatic progression in the terminal phase, the most appropriate intervention would have been early transition to palliative care with aggressive symptom management.

### 3.3 The expression characteristics of the RMS gene and the implications for this case: limitations of the era

Modern RMS diagnosis and treatment have entered the era of genotyping. This patient was initially diagnosed in 2009, when genetic testing had not yet been widely adopted in clinical practice, thus lacking crucial data such as Forkhead Box O1(FOXO1) fusion status, MYOD1 mutations, and Transcription Factor CP2(TFCP2) rearrangements. This absence itself reflects the evolution of clinical practice and also suggests the following important clinical implications:

FOXO1 fusion status: Paired Box 3/7-Forkhead Box O1 fusion (PAX3/7-FOXO1) fusion is present in about 80% of alveolar RMS, and the prognosis of these patients is significantly worse than that of fusion-negative patients (23). In this case, if the fusion detection can be performed at the initial diagnosis, it may be more accurate to predict the disease process and guide the treatment intensity.

MYOD1 mutation: In adult spindle cell/sclerotic RMS, the incidence of MYOD1 mutation is as high as 41%. The p.L122R mutation occurs in the conserved DNA- binding domain, resulting in transcriptional activation and MYC-like function. This suggests a key role in tumor development and is associated with extremely aggressive behavior and rapid progression (24). The patient has experienced multiple recurrences and resistance to various chemotherapy regimens, indicating the possible presence of such high-risk molecular abnormalities.

TFCP2 rearrangement: There is a specific gene fusion phenomenon in spindle cells and sclerosing rhabdomyosarcoma of the head and neck. If fused in sarcoma (FUS)- transcription factor CP2 (TFCP2) or Ewing sarcoma breakpoint region 1(EWSR1)-TFCP2 fusion is present in some cases, these tumors often show mixed epithelioid and spindle morphology. Immunohistochemistry shows co-expression of myogenic markers, Anaplastic Lymphoma Kinase (ALK) and cytokeratin (4), which provides a potential opportunity for targeted therapy.

The tortuous course of this case strongly suggests that all newly diagnosed RMS patients should be given comprehensive molecular detection as soon as possible, which not only helps to judge the prognosis, but also provides a direction for subsequent treatment, especially after the failure of traditional treatment options.

### 3.4 Cumulative toxicity of multimodal therapy: a reflection on clinical management strategies

The patient in this case received extremely complex multimodal treatment in 16 years, and its late facial cavity can be regarded as a concentrated expression of the cumulative effect of treatment-related toxicity. This complication prompts us to reflect on several key issues in the long-term management of RMS:

#### 3.4.1 Optimization of radiotherapy strategy: the treatment bottleneck reflected in this case

Although the patient received two courses of radiotherapy, which are necessary to control local diseases, this treatment also directly leads to the accumulation of damage to normal tissues. Retrospective assessment of the radiotherapy quality reveals several aspects that, by contemporary standards, would be considered suboptimal and contributory to the development of ultra-late complications. First, the cumulative radiation dose of approximately 84 Gy far exceeds the tolerance of maxillofacial bone and soft tissue; the TD5/5 for mandibular and maxillary bone is approximately 60–65 Gy, with significantly higher risk above 70 Gy (25). Second, the two courses were delivered within a relatively short time frame, precluding adequate sublethal damage repair and vascular recovery between treatment cycles. Third, the surgical interventions—including operations with ligation of the external carotid artery—compromised the already impaired regional blood supply, synergistically amplifying the radiation-induced microvascular injury.

Modern radiotherapy techniques have evolved substantially since this patient’s initial treatment in 2009-2013. Intensity-modulated radiotherapy (IMRT) and volumetric modulated arc therapy (VMAT) enable highly conformal dose distributions with steep dose gradients, thereby reducing the volume of normal tissue receiving intermediate-to-high doses. More recently, proton beam therapy (PBT) has emerged as a particularly attractive option for head and neck RMS due to the Bragg peak effect, which allows for precise deposition of dose within the target while sparing distal tissues. Systematic reviews and meta-analyses of PBT for RMS have reported 5-year local control rates of 84%, with acute and late toxicity rates of 9% and 26%, respectively—substantially lower than the 48-65% late toxicity rates observed with conventional photon therapy in parameningeal sites (21). Furthermore, long-term follow-up data from the Cooperative Weichteilsarkom Studiengruppe (CWS) trials demonstrate that cosmetic and functional defects are less frequently reported after proton therapy compared with conventional fractionated photon RT or hyperfractionated accelerated RT (22). For young patients with anticipated long survival durations, such as the present case, PBT should be strongly considered whenever feasible to minimize the lifelong risk of devastating late complications.

Prevention of radiation-induced osteonecrosis and soft tissue necrosis requires a multidisciplinary, proactive approach. The 2024 ISOO-MASCC-ASCO guideline for the prevention and management of osteoradionecrosis provides evidence-based recommendations that are highly relevant to this case. Key preventive strategies include: (1) pre-radiotherapy dental assessment and extraction of non-restorable teeth at least 2–3 weeks before initiating RT; (2) meticulous maintenance of oral hygiene throughout and after treatment; (3) avoidance of invasive dental or surgical procedures within the high-dose radiation field whenever possible; (4) smoking cessation and optimization of nutritional status; and (5) dose-volume planning with strict constraints for mandibular and maxillary bone (e.g., V60 Gy < 20% and mean dose < 45 Gy where clinically achievable) (25). For patients requiring re-irradiation, accumulating evidence suggests that cumulative doses to the mandible exceeding 100–120 Gy markedly elevate ORN risk. A 2025 retrospective study reported ORN in re-irradiated patients with mandibular cumulative doses of 128 Gy and 132 Gy (26). This case suggests that for young patients who may have a longer expected survival period, the target volume and dose should be carefully planned prospectively at the time of initial radiotherapy to preserve space and tissue tolerance for potential future re-irradiation (27). In the present case, none of these preventive measures were systematically implemented, reflecting both the era of treatment and the overwhelming focus on tumor control in a repeatedly relapsing patient.

#### 3.4.2 Dilemma and hope of systematic treatment: key insights from this case

In this case, patients failed to achieve long-term efficacy of multi-line treatment regimens including anlotinib, reflecting the bottleneck of adult RMS system treatment. In recent years, some new therapeutic strategies are being explored:

Immunotherapy: Studies have shown that chimeric antigen receptor (CAR) -T cells have the potential to inhibit tumor cell proliferation and growth when treating RMS with Ephrin type B receptor 4 (EPHB4) *in vitro* (28). In addition, a study of 134 patients with advanced sarcoma showed that programmed cell death protein 1(PD-1) inhibitors, such as nivolumab, also showed certain efficacy in the treatment of advanced RMS, with an objective remission rate of 16% and a long-term benefit trend (29).

Targeted therapy: drugs for specific molecular abnormalities, such as ALK inhibitors for TFCP2 rearrangement RMS, or Fibroblast growth factor receptor 4 (FGFR4) inhibitors for patients with corresponding mutations, may provide new options for patients who have failed traditional chemotherapy (30).

#### 3.4.3 The change of diagnosis and treatment concept from ‘ tumor control ‘ to ‘ quality of life ‘: the core revelation of this case

The core implication of this case may be that for patients with advanced RMS who have undergone years of treatment, when the time is appropriate, the treatment goal should shift from pursuing tumor control to optimizing quality of life and dealing with symptoms. The patient ‘s decision to reject gastrostomy should be respected after full communication and alternative support options should be sought.

#### 3.4.4 Management strategies for established radiation necrosis complications: evidence-based options and their relevance to this case

Once severe radiation-induced tissue necrosis and maxillofacial cavity have developed, therapeutic options are limited and outcomes are generally poor. A structured, evidence-based management approach is essential, tailored to the extent of disease, patient performance status, and treatment goals. The 2024 ISOO-MASCC-ASCO guideline provides evidence-based recommendations on ORN prevention and management, and its recommendations provide a valuable framework for reflecting on what might have been attempted in this case had the complication been recognized earlier or had the patient presented with a more favorable performance status (25).

For early-stage radiation necrosis (confined to alveolar bone or small soft tissue defects without full-thickness involvement), conservative management is the first-line approach. This includes meticulous local wound care with antiseptic irrigations, systemic antibiotics for clinically evident infection, appropriate analgesia, and nutritional support (25). Medical management with pentoxifylline-based regimens has shown efficacy in mandibular osteoradionecrosis, with a retrospective cohort study of 169 patients reporting a 54.4% healing rate after a mean of 12.9 months; notably, the PENTO regimen (without clodronate) was significantly superior to PENTOCLO (p = 0.0001), and outcomes were most favorable in Notani stage I and non-infected cases (31). For this patient, however, the extensive full-thickness soft tissue and bone defect with exposure of the skull base placed her far beyond the scope of conservative or medical management alone.

Hyperbaric oxygen therapy (HBOT) has historically been advocated for ORN, but recent high-level evidence has challenged its routine use. Combined data from two randomized trials (DAHANCA-21 and NWHHT2009-1) showed that HBOT did not significantly improve healing after surgical removal of necrotic bone compared with standard care alone (70% versus 51%; not statistically significant due to insufficient power) (32). The 2024 ISOO-MASCC-ASCO guideline therefore concluded that the use of HBOT in ORN prevention and management remains largely unjustified, with limited evidence to support its practice (25). For this patient, given the extensive soft tissue loss and full-thickness necrosis extending to the skull base, HBOT would have been unlikely to alter the clinical course meaningfully.

Surgical management remains the definitive intervention for advanced radiation necrosis with full-thickness tissue loss. Contemporary staging systems, such as the Notani classification and the recently proposed ClinRad classification by Watson et al., facilitate clinical decision-making by stratifying disease severity according to the vertical extent of bone necrosis and the presence or absence of exposed bone or fistula (33). For maxillary and midfacial ORN—anatomically distinct from the more commonly reported mandibular ORN—reconstructive options include local advancement flaps, pedicled flaps (e.g., temporalis muscle flap), and free tissue transfer (e.g., radial forearm free flap, anterolateral thigh flap, or rectus abdominis flap). In cases with extensive maxillectomy defects or skull base exposure, vascularized bone grafts (e.g., fibula or iliac crest) may be required to restore facial contour and provide structural support. However, free flap reconstruction in heavily irradiated fields carries substantial risks, including partial or complete flap loss due to compromised recipient vessel quality, delayed wound healing, and persistent infection (34). In this case, the combination of extensive radiation-induced vascular damage, poor nutritional status (BMI 18.1 kg/m²), and the patient’s unwillingness to undergo further invasive procedures precluded surgical reconstruction.

In retrospect, the most impactful intervention for this patient would have been prevention through modern radiotherapy planning and early, aggressive management of the initial soft tissue ulceration before progression to full-thickness necrosis. For clinicians managing similar long-term survivors, the key lessons are: (1) maintain a high index of suspicion for late radiation injury in any new local symptoms, regardless of latency; (2) implement preventive strategies (dental care, dose constraints, smoking cessation) before initiating radiotherapy; (3) recognize early signs of ORN (pain, mucosal breakdown, exposed bone) and intervene promptly with conservative measures or PENTOCLO before irreversible full-thickness destruction occurs; and (4) establish a lifelong, multidisciplinary follow-up system integrating oncology, maxillofacial surgery, dental medicine, and palliative care to optimize both disease surveillance and quality of life (25, 31).

### 3.5 Clinical enlightenment and prospect

This case report has several limitations that require clarification. The primary limitation lies in the absence of pathological confirmation of the cavity. Although clinical and imaging features, such as the lack of significant enhancement signal within the cavity on MRI, do not support active tumor recurrence and are more consistent with a diagnosis of radiation necrosis, the possibility of the presence of indolent tumor cells or mixed pathological components cannot be completely excluded. The decision not to perform biopsy was based on the patient’s frail condition at the time, and the treatment goal had shifted from tumor eradication to symptom control and maintenance of quality of life. Furthermore, the classification and staging of osteoradionecrosis have advanced considerably. Watson et al. developed and standardized a risk-based ORN classification system that stratifies patients according to clinical and imaging characteristics, enabling more precise prognostication and treatment allocation (33). This system, alongside the established Notani classification and the contemporary staging proposed by Fritz et al., provides clinicians with robust tools to categorize disease severity and select appropriate interventions—from conservative measures for early-stage disease to aggressive debridement and vascularized reconstruction for advanced, full-thickness necrosis (34).

Despite this limitation, the value of this case lies in its revelation that for long-term survivors of rhabdomyosarcoma who have undergone high-dose radiotherapy, the risk of severe late radiation injury may persist for decades, rather than being limited to the early stages after treatment. This finding necessitates clinicians to adopt a long-term historical perspective when facing any new local symptoms in such patients, and to prioritize late treatment toxicity in their differential diagnosis. Looking ahead, with advancements in radiotherapy techniques (such as proton therapy), it is expected that the risk of such devastating late complications can be reduced at their source through more precise dose control. Simultaneously, establishing a lifelong follow-up system for long-term survivors of rhabdomyosarcoma is crucial for early detection and management of such issues. Ultimately, with the advancement of molecular diagnostic techniques and the development of new strategies such as targeted therapy and immunotherapy, we anticipate achieving more precise and individualized management of rhabdomyosarcoma. This will minimize treatment toxicity while controlling the disease, truly improving patients’ long-term quality of life. The importance of long-term, structured follow-up for RMS survivors cannot be overstated. Current survivorship guidelines recommend clinical and imaging surveillance with particular attention to local recurrence, secondary malignancies, and late treatment toxicities. For patients who received high-dose radiotherapy to the head and neck, this follow-up should be extended indefinitely and should incorporate dental examinations, nutritional assessments, and early referral to maxillofacial specialists for any signs of mucosal breakdown, pain, or exposed bone (25). The development of patient-reported outcome measures specific to head and neck cancer survivorship, alongside biomarker research to identify individuals at heightened genetic risk for late radiation toxicity, represents promising avenues for personalized risk stratification and early intervention.

## 4 Conclusions

This case highlights the importance of prioritizing multimodal treatment in the long-term management of adult head and face rhabdomyosarcoma (RMS), with particular attention to the delayed and cumulative toxicities of radiotherapy. For long-term survivors undergoing complex treatments, clinical decision-making should, at the appropriate time, shift focus from aggressive anti-tumor therapy to managing complications and optimizing quality of life. This case also underscores the necessity of integrating modern radiotherapy quality standards—including dose constraints, conformal techniques, and normal tissue sparing principles—into the initial treatment planning for all patients with head and neck RMS. For those who develop established radiation necrosis, a multidisciplinary team approach involving medical oncology, radiation oncology, maxillofacial surgery, and palliative care is essential to navigate the limited but evolving therapeutic landscape, which now includes structured conservative protocols, medical therapy with PENTOCLO, and advanced reconstructive surgery. Ultimately, the goal must be to prevent such catastrophic late complications through prospective planning, and when they do occur, to recognize them early and manage them aggressively within an evidence-based, patient-centered framework.

## Data Availability

All data associated with this case report are openly available in the article. The patient has provided written informed consent for the publication of their clinical details and accompanying images. No additional unpublished data are available.
